# Metabolomics in spondylarthritis

**DOI:** 10.1186/s41927-025-00546-3

**Published:** 2025-07-23

**Authors:** Susann Patschan, Constantin Remus, Inga Claus, Meike Hoffmeister, Oliver Ritter, Daniel Patschan

**Affiliations:** 1 Department of Internal Medicine I, Cardiology, Nephrology and Internal Intensive Medicine, Brandenburg University Hospital, Brandenburg Medical School (Theodor Fontane), Brandenburg an der Havel, Germany; 2 Institute of Biochemistry, Brandenburg Medical School (Theodor Fontane), Brandenburg an der Havel, Germany; 3 Faculty of Health Sciences (FGW), Joint Faculty of the University of Potsdam, The Brandenburg Medical School Theodor Fontane and the Brandenburg Technical University Cottbus-Senftenberg, Cottbus, Germany

**Keywords:** SpA, Disease activity, Cardiovascular risk, Metabolomics

## Abstract

**Background and aim:**

Spondyloarthritides (SpA) are common entities of the inflammatory rheumatic type. There are still 3 relevant problems in everyday clinical practice: early disease detection, cardiovascular risk assessment, and less so, disease activity measurement. Metabolomics allows the quantification of a large number of small-molecule substances from biological samples.

**Methods:**

The following databases were searched for references: PubMed, Web of Science, Cochrane Library, Scopus. The period lasted from 1973 until 2024.

**Results:**

Finally, 14 analyses were identified. Most studies have evaluated patients with established disease. Some studies were able to un-mask metabolomic characteristics of certain forms of SpA. Approaches that utilize an integrative view of several metabolites in combination with general patient characteristics appear to be quite promising. Such approaches are suitable, for example, for assessing activity in psoriatic arthritis (PsA) or evaluating cardiovascular risk in individuals with psoriatic disease.

**Conclusions:**

Metabolomics are helpful in identifying new diagnostic and predictive parameters in SpA, so far mainly in PsA. An almost consistent limitation of the studies to date is the inclusion of patients with already manifest disease.

## Introduction

Seronegative spondyloarthropathies or spondyloarthritides (SpA) are represented by the following entities: ankylosing spondylitis (AS), psoriatic arthritis (PsA), reactive arthritis, enteropathic arthritis (EPA), anterior uveitis and undifferentiated spondyloarthritis (unSpA) [1, 2]. The prevalence of the disease group roughly corresponds to the prevalence of rheumatoid arthritis (RA) [3] as the most common isolated disease of the inflammatory rheumatic group [4]. SpA are variably characterised by inflammatory back pain or a mostly asymmetrical oligoarthritis of larger peripheral joints [5]. Optional organ complications occur with varying frequency in patients, including more common issues such as iritis and significant skin changes, and less common manifestations like pleuritis, pericarditis, or renal complications (e.g., IgA nephritis in ankylosing spondylitis) [6]. Depending on the clinical involvement of the musculoskeletal system, a distinction is made between axial and peripheral forms [2, 7]. The axial form can be further categorized into radiographic (with evidence of structural changes in the spine on conventional imaging) and non-radiographic (without such evidence) [8]. Patients with spondyloarthritis (SpA) are also at an increased risk of cardiovascular disease, a trend that is seen in individuals with other inflammatory rheumatic diseases [9]. This heightened cardiovascular morbidity is partly due to the proatherogenic effects of medications used to manage disease activity and progression, particularly nonsteroidal anti-inflammatory drugs (NSAIDs) [10]. Additionally, it is a direct consequence of the inflammatory activity associated with the underlying diseases [9].

The recommendations for the specific treatment of the various forms of spondyloarthritis (SpA) are heterogeneous; however, they share comparable goals: reducing painful functional impairments and preserving joint function and structure. A third objective may be the reduction of cardiovascular risk, for which specific guidelines have already been published [11]. Although numerous, in some cases specific, active substances are now available for the management of SpA, predicting the therapeutic response in individual cases is difficult. Furthermore, established assessment tools for cardiovascular risk stratification do not adequately capture the increase in risk in inflammatory rheumatic diseases. Therefore, the search for new diagnostic strategies to assess therapy susceptibility and cardiovascular risk continues.

Metabolomics studies offer a possible approach here. The term metabolomics describes the detection and quantification of small-molecule ( < 1.5 kD) substances from biological samples, usually blood or tissue samples. Additional types of “−omics” analyses have been established in recent years, such as genomics, epigenomics, and proteomics. These advancements enable researchers to gain more sophisticated insights into the complexity of biological processes.

There have already been excellent review articles published on this topic. Notably, a paper by Huang and colleagues from 2022 [12] provides a compelling summary of data regarding deviations in amino acid, lipid, and carbohydrate metabolism across various entities of spondyloarthritis (SpA). The article also compares SpA with other conditions within the spectrum of inflammatory rheumatic diseases. In contrast, our article focuses on metabolomics for the identification of diagnostic and prognostic markers, and indicators of disease activity and (cardiovascular) complications.

## Methods

**Fig. 1:**
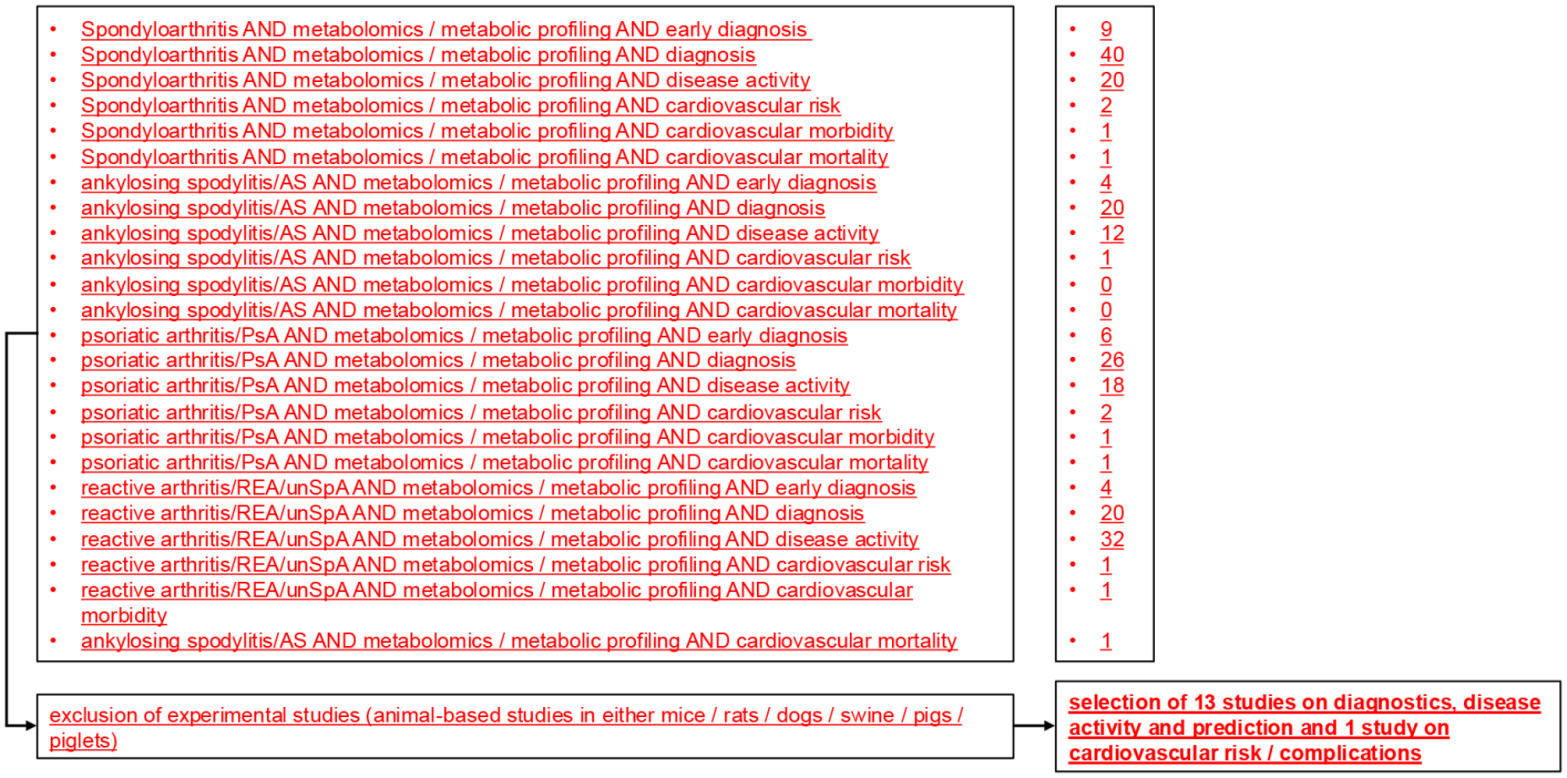
Search approach and the number of references obtained

The following databases were searched for references: PubMed, Web of Science, Cochrane Library, Scopus. The period lasted from 1973 until 2024. The following terms were variably utilized: ´spondyloathritis´, ´seronegative spondyloarthritis´, ´ankylosing spondylitis´, ´AS´, ´arthritis psoriatica´, ´psoriatic arthritis´, ´PsA´, ´undifferentiated spondyloarthritis´, ´unSpA´, ´reactive arthritis´, ´REA´, ´metabolomics´, ´metabolic profiling´, ´early diagnosis´, ´diagnosis´, ´early disease´, ´disease activity´, ´cardiovascular risk´,´cardiovascular morbidity´, ´cardiovascular mortality´. Please refer to Fig. 1 for a summary of the search approach and the number of references obtained.

Selected articles are discussed according to the date of publication (older to newer), unless otherwise stated. The methodological approaches utilized in the context of metabolic profiling are mentioned only sporadically in the text. For information regarding the technical specifics (capabilities and limitations) of the various spectroscopic procedures, please refer to relevant review articles [13, 14].

### Identification of diagnostic, prognostic and disease activity markers

#### Diagnostics

A logistically complex study involved 44 patients [15] with ankylosing spondylitis (AS) and 44 healthy controls, along with an additional group of 30 patients with AS and 30 with femoral neck fractures (FNF) for tissue sample analysis. After applying inclusion and exclusion criteria, 5 AS patients with comorbidities were excluded. The demographics of the AS and control groups were comparable in terms of age, sex, and BMI. Using 1 H NMR spectroscopy, the study analyzed plasma, urine, and tissue samples. The analysis revealed that urine, plasma, and polar tissue extracts primarily contained amino acids, glucose, and lipids, while non-polar tissue extracts were mainly composed of triglycerides. Principal Component Analysis (PCA) indicated significant differences in tissue samples between AS patients and controls, with plasma samples showing some discrimination but urine samples lacking complete distinction. Partial Least Squares Discriminant Analysis (PLS-DA) and Orthogonal PLS-DA (OPLS-DA) yielded high R2Y and Q2 values that demonstrated good model discrimination and predictive ability. Although urine samples had lower Q2 values, they were still deemed acceptable. Cross-validation tests confirmed that the models were not over-fitted. From the multivariate analysis, 20 potential biomarkers for AS were identified across plasma, urine, and tissue samples. These metabolites were linked to various metabolic pathways, including fat metabolism, glucose metabolism, and immune regulation. However, no individual marker could be identified as a new indicator of early disease, as all included patients were already suffering from established AS.

A study published in 2017 [16] involved 20 patients with ankylosing spondylitis (AS) and 20 healthy controls (HCs). Both groups were matched for age and gender, with significant differences in male representation and mean age (35.2 years for AS vs. 36.68 years for HCs). Patients exhibited increased disease activity as indicated by higher BASDAI, ASDAS-CRP, ESR, and CPR scores. A total of 995 metabolites were identified in serum samples, and principal component analysis (PCA) indicated that the AS group could be distinguished from HCs using an OPLS-DA model, which showed good discrimination (R2Y = 0.987, Q2Y = 0.779). Sixty-one differential metabolites were identified as potential biomarkers, with 19 significantly increased and 42 significantly decreased in AS patients. Bioinformatics analysis revealed that these metabolites were involved in 27 metabolic pathways, notably glycerophospholipid and sphingolipid metabolism. Receiver operating characteristic (ROC) curve analysis showed that 57 metabolites had diagnostic performance with area under the curve (AUC) values between 0.7 and 0.9. A Random Forest (RF) model achieved 95% predictive accuracy, distinguishing AS patients from HCs effectively. The top metabolites identified included various amino acids, cofactors, and lipids. To refine the diagnostic model, LASSO regression was employed, resulting in a final model comprising nine metabolites, including cysteinylglycine disulfide and choline, with an AUC of 1, indicating excellent diagnostic capability for AS. The potential for early detection of AS through the use of the aforementioned metabolites is a subject that requires further evaluation; however, the preliminary results are highly encouraging.

In 2018, Dubey and colleagues [17] addressed a very specific aspect of rheumatological medicine: the differentiation between reactive arthritis and undifferentiated spondyloarthritis (unSpA) from rheumatoid arthritis. Undifferentiated SpA is defined as those forms of spondyloarthritis that do not meet the diagnostic criteria for ankylosing spondylitis, psoriatic arthritis, or enteropathic arthritis, and are not triggered by an obvious infection [18]. In the study, patients with reactive arthritis and undifferentiated spondyloarthritis were grouped together as reactive arthritis (REA). This group included 52 individuals, who were compared to 29 rheumatoid arthritis (RA) patients and 82 healthy controls. The healthy controls were further divided into two age categories: younger (mean age 32.7 ± 7.5 years) and older (mean age 41.2 ± 9.2 years). Using NMR-based metabolomics, 4 (or five) metabolites were identified that differentiated between patients with reactive arthritis (REA) and rheumatoid arthritis (RA) based on concentration differences: valine, leucine, arginine, lysine, and phenylalanine. All substances were found to be at higher concentrations in reactive arthritis (REA), and these deviations were attributed to different autoimmune processes in reactive arthritis and undifferentiated spondyloarthritis compared to rheumatoid arthritis.

Another study, published in 2019 [19] analyzed paired sera and synovial fluid from 32 patients, comprising 19 with reactive arthritis (ReA) and 13 with undifferentiated spondyloarthritis (SpA). Among the ReA patients, 6 experienced inflammatory backache, and 13 met criteria for peripheral SpA, but were classified solely as ReA. The cohort included 22% women, with a median age of 26 years. Clinical presentations varied, with 12% having a first episode of illness, 64% experiencing relapses, and 24% showing persistent arthritis. Articular involvement was predominantly oligoarticular (59%). The metabolomic analysis revealed distinct profiles in patient sera compared to healthy controls, with significant differences in metabolites such as malonate, pyruvate, and phenylalanine. Additionally, a comparison between sera and synovial fluid showed notable differences in metabolites, with pyruvate and acetoacetate being higher in synovial fluid. Pathway analysis identified five significant pathways in the patient sera versus controls and similar findings in the synovial fluid. Importantly, no differences were observed in the metabolomic profiles between ReA and undifferentiated pSpA, nor between HLA-B27 positive and negative patients, suggesting that these factors do not influence the metabolic profiles in this context. These findings were interpreted positively, suggesting that there appear to be few pathogenic differences between REA and unSpA.

Muhammed and colleagues [20] once again focused on the differentiation between REA/unSpA and RA and osteoarthritis. In this context, targeted metabolomics was conducted. Targeted analyses are less about screening a large number of candidate molecules and more about investigating the potential suitability or involvement of specific substrates as biomarkers or in pathophysiological processes. In this study, the phenylalanine/tyrosine ratio in synovial fluid and serum was quantified. Patients with REA/unSpA exhibited a higher synovial ratio compared to the other two entities. Furthermore, the ratio in the synovial fluid was significantly higher than that in the serum. Ultimately, it was proposed as a specific indicator for REA/unSpA.

Souto-Carneiro et al. [21] conducted an untargeted analysis aimed at identifying differences between seronegative rheumatoid arthritis (negRA) and psoriatic arthritis (PsA). In clinical practice, it is often difficult to clearly distinguish between these two entities, especially when PsA is not associated with HLA-B27 and exhibits a similar pattern of joint involvement as RA. Approximately 50 years ago, Moll and Wright [22] summarized the possible manifestation patterns of PsA. On one hand, the investigation revealed differing serum concentrations of various amino acids; on the other hand, there were notable abnormalities in the ratios of specific serum lipids. Both the sensitivity and specificity of the lipid ratios improved (from 70% to 84.5%) when a multivariate model was applied, taking into account covariates such as age, gender, and the concentrations of alanine, succinate, and creatine phosphate. In any case, the study demonstrated a potential option for differentiating between the entities of psoriatic arthritis (PsA) and seronegative rheumatoid arthritis (negRA), although the analyzed group sizes were relatively small (PsA 73, negRA 49).

A study published in 2021 [23] examined REA with a sample size of 58 participants, comparing them to groups consisting of 21 individuals with rheumatoid arthritis and 20 individuals with osteoarthritis. Synovial fluid was once again used for metabolic profiling. In comparison to rheumatoid arthritis, two substances showed different concentrations in reactive arthritis (alanine and carnitine). When compared to osteoarthritis, there were even six differences: N-acetylglucosamine (NAG), glutamate, glycerol, isoleucine, alanine, and glucose. The study highlights the metabolic characteristics of reactive arthritis, which could be particularly helpful in differentiating it from degenerative joint damage through synovial analysis. Reactive arthritis is primarily characterized by an oligoarticular pattern of involvement, making it clinically challenging at times to distinguish from active knee or hip osteoarthritis.

Another study on psoriatic diesease was also published in 2021 [24]. Although the target population was different (healthy controls, patients with psoriasis without arthritis, and those with arthritis), the study aimed, like the one by Gupta et al. [26] to identify metabolite differences between two forms of one disease (psoriasis with versus without psoriatic arthritis). Some psoriasis patients developed psoriatic arthritis (PsA) over time, and individuals with PsA were categorized based on disease activity (mild, moderate, severe). Initially, the serum metabolome did not show significant differences between psoriasis patients without PsA, those with newly developed PsA, and those with pre-existing mild PsA. In contrast, patients with severe PsA exhibited elevated levels of specific long-chain fatty acids. Additionally, certain eicosanoids, which have variable pro- and anti-inflammatory effects, were detected exclusively in the serum of patients with moderate to severe PsA activity. The study highlights that highly active PsA courses are characterized by additional disturbances in lipid metabolism, which could potentially be utilized for differential diagnosis. Whether these changes are causal or reactive, however, remains unclear.

A very recent study published by our group [25] involved 100 patients (66 women, 34 men) with a mean age of 54.2 years, alongside a control group of 164 individuals (65% women, 35% men) with a slightly lower average age of 46.8 years. Among the patients, 50 had ankylosing spondylitis (AS) and 50 had psoriatic arthritis (PsA), with average disease durations of 14.5 years for AS and 9.7 years for PsA. The HLA-B27 test was positive in 88% of AS patients and 27% of PsA patients, with sacroiliitis diagnosed in 86% of AS cases and 14% of PsA cases. Metabolite analysis revealed significant differences between spondyloarthritis (SpA) patients and healthy controls, with 26 metabolites being lower and 9 elevated in the SpA group. Comparisons between AS and controls showed lower concentrations of many metabolites in AS patients, while specific metabolites such as acetoacetic acid and myo-inositol were significantly elevated. In contrast, PsA patients exhibited 11 metabolites at lower concentrations and 19 elevated compared to controls. Notably, glucose and glycerol levels were significantly lower in AS compared to PsA. We also explored various risk factors and morbidities, such as obesity, alcohol consumption, and hypertension, but found no significant differences in metabolite concentrations linked to these factors in either AS or PsA patients. However, patients with obesity displayed lower levels of certain amino acids compared to those with normal weight, and hypertensive SpA patients had higher levels of myo-inositol and glucose compared to normotensive patients. We have concluded that both ankylosing spondylitis (AS) and psoriatic arthritis (PsA) are characterized by several alterations in amino acid and lipid metabolism. The deviations observed in hypertensive and overweight patients with spondyloarthritis (SpA) could have potential implications for future cardiovascular risk assessment, although the study design does not allow for definitive conclusions in this regard.

#### Prognosis and disease activity

In 2012, Fischer and colleagues [26] analyzed a total of 18 patients with ankylosing spondylitis (AS) and 9 healthy individuals, employing both metabolic profiling and proteomics. The proteomic analysis revealed 22 proteins that were distinctly concentrated in AS patients compared to healthy controls. At the metabolic level, the study identified 7,000 molecular characteristics, with particularly notable findings related to vitamin D metabolism. One striking observation was the deviation in the metabolite (23S,25 R)-25-hydroxyvitamin D3 26,23-peroxylactone. Additionally, the ratio of this metabolite to the vitamin D binding protein was abnormal, leading to the conclusion that disturbances in vitamin D homeostasis may contribute to bone damage in AS. While these findings do not necessarily imply immediate advancements in the diagnostic capabilities for AS, they suggest that significant disorders in vitamin D metabolism could be linked to an increased risk of disease progression. This observation could be of prognostic significance.

In a study published in 2021 [27], metabolic profiling was conducted in patients with peripheral and axial spondyloarthritis (SpA), who were also characterized by varying levels of disease activity. Ultimately, 86 controls and 81 patients with SpA were included, with serum samples used for the metabolic analyses. In contrast to other studies, this analysis followed up on some patients and conducted additional examinations at a later time point. Initially, patients with spondyloarthritis (SpA) exhibited higher serum concentrations of several metabolites, including various amino acids, acetate, choline, N-acetyl glycoproteins, Nα-acetyl lysine, and creatine/creatinine, among others. Conversely, reduced concentrations were observed for low- and very low-density lipoproteins, as well as polyunsaturated lipids. Additionally, there were differences between individuals with axial and peripheral SpA, as well as among individuals with varying levels of disease activity. The study demonstrated that metabolomics can indeed be of diagnostic value, not only in differentiating one condition from others but also in daily clinical management, such as assessing disease activity and monitoring treatment response. The study substantially helps to establish biomarkers to differentiate between axial and peripheral SpA.

Another study related to PsA was published in 2023 [28]. The metabolome of psoriasis patients with and without arthritis (PsA) was analyzed again, not just at a single point in time, but longitudinally, both before and after the onset of PsA. In contrast to the previous study, however, the PsA activity level was not taken into account. In any case, there were significant differences between healthy controls, Ps and PsA in the concentrations of various serum metabolites, namely bile acids, purines, pyrimidines and others. Specifically, bile acids and butyrate showed reduced concentrations in PS individuals with newly manifested PsA. On the other hand, elevated levels of leukotriene B4 and glycoursodeoxycholic acid sulfate were identified as specific predictors of PsA manifestation. About 25% of all Ps patients are affected by PsA during the course of the disease [29] and the prediction of arthritis is still an almost unsolved problem in dermatologic/rheumatologic medicine. Therefore, the study is absolutely valuable.

A new study from 2024 [30] was dedicated to the identification of PsA activity markers, with reference to the difficulties of reliably determining the activity of psoriatic arthritis. Included patients were assigned to one of three clinical activity groups, based on the psoriatic arthritis disease activity score (PASDAS). The results of the metabolic analyses were evaluated using machine learning methods. Although it is not possible to go into the details of the individual algorithms here, they should be briefly mentioned: adaptive boosting (AdaBoost), decision tree (J48), LogitBoost, logistic regression, logistic regression with L1 (Lasso) regularization, logistic regression with L2 (Ridge) regularization, Naïve Bayes, random forest and support vector machine (SVM), as implemented in the mlr 2.15.0 package (Bischl B). Effectiveness was ultimately measured using the AUC. The focus of metabolomics was primarily on lipid metabolites. Using the LogitBoost model, low and high disease activity could be differentiated most reliably, the AUC was 0.818. Further AUC values were 0.74 (differentiation between moderate and high activity) and 0.76 (low versus moderate activity). Among other metabolites, the following have been identified as particularly potent in differentiating between low and high disease activity: hippuric acid, DL-tryptophan, sphingomyelin, 4-acetamidobenzoic acid, and N-phenylacetylglutamine. The study impressively demonstrates the diagnostic possibilities offered by the simultaneous analysis of different metabolites and subsequent algorithm-based data evaluation. Such approaches could possibly gain in importance in the future.

#### Identification of cardiovascular disease or complication markers

Very few studies address cardiovascular morbidity or risk markers in SpA. Strictly speaking, only one study from 2021 could be identified [31]. The study was carried out primarily due to the limited validity of conventional CV risk prediction in rheumatics using, for example, the Framingham score or the recently updated ESC recommendations. Patients with psoriatic diseases were examined, the term was used collectively for psoriasis and arthritis psoriatica. From a total collective of 977 individuals, a cardiovascular event was identified in 70 patients. Various metabolites were associated with either increased (glycoprotein acetyls, apolipoprotein B and cholesterol remnants) or decreased (alanine, tyrosine, degree of unsaturation of fatty acids and high-density lipoprotein particles) CVR. A prediction model extended to include selected metabolites, which also took gender and age into account, resulted in an AUC of 0.79 for cardiovascular events. However, when 11 metabolites were added to the Framingham score in a prediction model, the predictive power did not increase significantly (AUC 0,75 versus 0.73; *p* = 0.72). Although the study does not prove the complete unsuitability of the Framingham score for cardiovascular risk assessment of patients with psoriatic disease, it does show that the assessment of certain metabolites in addition to sex and age optimizes CVR prediction (See Table 1).

**Table 1:** Summary of the studies discussed in the text

reference	study design	results		significance	
**diagnostics, prognosis and disease activity**					
Wang et al., 2016 [15]	blood, urine and hip tissue samples from 30 AS patients; control group included		differences in 20 metabolites - lipid metabolism, intestinal microbial metabolism, glucose and choline metabolism		identification of AS-associated metabolic abberations, no recognition of biomarker of early disease
Li et al., 2017 [16]	comparison of 20 AS patients and 20 controls		incorporation of nine metabolites in a diagnostic model of AS		model potentially suitable for early AS diagnosis
Dubey et al., 2018 [17]	inclusion of RA (*n* = 29), REA, unSpA and controls (*n* = 82); REA and unSpA summarized in one group (*n* = 52)		serum valine, leucine, arginine, lysine, and phenylalanine higher in REA than in unSpA		metabolic deviations reflect different autoimmune processes in REA (REA and unSpA)
Ahmed et al., 2019 [19]	inclusion of 19 patients with REA, 13 patients with unSpA and 18 controls: analysis of serum and synovial fluid		significant metabolic differences between controls and REA/unSpA; comparable patterns in REA and SpA		pathogenic differences between REA and unSpA only minor
Muhammed et al., 2020 [20]	inclusion of REA, unSpA, RA and osteoarthritis; analysis of serum and synovial fluid		synovial phenylalanine/tyrosine ratio higher in REA/unSpA compared to RA and controls; synovial ratio higher than serum ratio		phenylalanine/tyrosine ratio as specific RA/unSpA indicator
Souto-Carneiro et al., 2020 [21]	patients with seronegative RA (negRA) and PsA		differences in various serum amoni acids and in several serum lipid ratios		metabolites allow to distinguish between negRA and PsA
Dubey et al., 2021 [23]	inclusion of REA (*n* = 58), RA (*n* = 21) and osteoarthritis (*n* = 20); analysis of synovial fluid		two differentially concentrated metabolites in REA as compared to RA (alanine and carnitine); six differences between REA and osteoathritis (N-acetylglu- cosamine (NAG), glutamate, glycerol, isoleucine, alanine, glucose)		metabolites allow discrimination between REA and osteoarthrits
Looby et al., 2021 [24]	inclusion of healthy controls, patients with psoriasis ±arthritis; some Ps patients developed PsA over time		elevated levels of specific long-chain fatty acids in patients with severe PsA; certain eicosanoids exclusively detected in patients with moderate to severe PsA activity		disturbances in lipid metabolism characterize highly active PsA
Remus et al., 2025 [25]	analysis of blood samples from 50 patients with AS, 50 patients with PsA and 164 controls		several metabolic differences bewteen AS and controls and between PsA and controls, only minor differences between AS and PsA (glucose and glycerol), obese and hypertensive SpA patients exhibit metabolic differences compared to normal-weight and non-hypertensive patients		metabolomic features associated with AS and PsA may be potentially useful for differential diagnosis, certain amino acids and myo-inositol could also be valuable for cardiovascular risk assessment
Fischer et al., 2012 [26]	AS (*n* = 18) versus controls (*n* = 9); proteomics and metabolomics analysis		22 differentially expressed proteins and over 7,000 aberrant metabolites in AS; disturbed vitamin D metabolism in AS		vitamin D disorders linked to AS progression
Gupta et al., 2021 [27]	SpA (axial and peripheral – *n* = 81) and controls (*n* = 86); analysis of serum with follow up in some individuals		differences in various amino acids, acetate, choline, N-acetyl glycoproteins, Nα-acetyl lysine, and creatine/creatinine and others between SpA and controls; differences between axial and peripheral SpA and between patients with different levels of disease activity		metabolic profiling potentially helpful in early discrimination between axial and peripheral types
Paine et al., 2023 [28]	Ps patients with and without PsA, analysis before and after PsA onset		identification of leukotriene B4 and glycoursodeoxycholic acid sulfate as specific predictors of PsA manifestation		identification of specific predictors of PsA manifestation
Koussiouris et al., 2024 [30]	PsA patients with different levels of activity according to the PASDAS; primary focus of metabolics analysis on lipid metabolism; integration of machine learning algorithms		LogitBoost model reliably differentiated between low and high disease activity		new model for disease activity assessment in PsA
**cardiovascular disease or complications**					
Colaco et al., 2021 [31]	inclusion of Ps and PsA (psoriatic disease)	identification a CVR prediction model that included selected metabolites, no increase in predictive power if 11 metabolites were added to the Framingham score		identification of a new CVR prediction model in Ps and PsA	

Abbreviations: AS – ankylosing spondylitis; REA – reactive arthritis; unSpA – undifferentiated spondyloarthritis; negRA – seronegative rhheumatoid arthritis; Ps – psoriasis; PsA – arthritis psoriatica; PASDAS - psoriatic arthritis disease activity

## Conclusive discussion

First, the key findings should be summarized:

- Undoubtedly, there are metabolic differences between healthy individuals and those with ankylosing spondylitis [15, 16, 25].
- Metabolomics enables the differentiation between reactive arthritis, undifferentiated spondyloarthritis, rheumatoid arthritis, and osteoarthritis [17, 19, 20].
- Metabolic profiling can distinguish between seronegative rheumatoid arthritis and psoriatic arthritis [21].
- The extent to which identified differences occur prior to the onset of the disease, thereby enabling early detection, remains to be determined.
- Regarding the suitability of metabolomic studies for identifying SpA activity markers, particular attention should be drawn to the recent approach utilizing machine learning [30].
- If cardiovascular manifestations are considered complications of distinct seronegative spondyloarthritis, as evidenced by the publication of a EULAR guideline in 2015 on cardiovascular risk management for RA and related conditions [11], the data regarding new biomarkers remains quitey limited. One study [31] suggests that incorporating metabolomic findings, in addition to general variables such as age and gender, can enhance the prediction of cardiovascular risk.

A fundamental challenge with metabolomic analyses, not only in individuals with inflammatory rheumatic diseases, is the acquisition of extensive datasets that may include concentration changes of over 100 metabolites. Interpreting such large volumes of data is anything but straightforward. In most cases, metabolomic studies reveal deviations in profiles such as amino acids or lipids, among other metabolites. A review of the metabolomic literature indicates that such deviations occur across a wide range of diseases. Based on our own experience, we can reference the body of data related to acute kidney injury, for which our group has authored two review articles [32, 33]. Identifying disease-specific patterns during these analyses is a rare occurrence. For instance, in the case of acute kidney injury, various tryptophan metabolites (kynurenine (KYN), 5-hydroxyindoleacetic acid (5-HIAA), indoxyl sulfate (IS)) were identified as potential biomarkers for acute and chronic kidney disease [34]. However, such specific findings remain the exception rather than the rule. Are there any specific or at least typical metabolomic findings associated with spondyloarthritis (SpA) that can be identified? For instance, the increased phenylalanine/tyrosine ratio in synovial fluid and serum in patients with reactive arthritis or undifferentiated SpA compared to those with osteoarthritis could be considered a more specific finding [20]. Another relevant finding in this context is the elevated levels of leukotriene B4 and glycoursodeoxycholic acid sulfate in patients with psoriasis. Both substrates have been identified as specific predictors of psoriatic arthritis [28]. This finding stands in contrast to the study by Li et al. [16], which identified a total of 995 metabolites in varying concentrations between AS patients and healthy individuals.

One unresolved issue remains the early detection of diseases. In the cited study by Paine and colleagues [28], the identification of the predictive properties of leukotriene B4 and glycocholic acid sulfate was achieved in a cohort of patients with psoriasis, a group known to have an approximately 25% risk of developing psoriatic arthritis (PsA) [29]. This allowed the authors to focus on a high-risk population. However, a similar approach is more challenging for ankylosing spondylitis (AS), which, although characterized by a distinct genetic predisposition, does not consistently or reliably occur within families. Even fewer options for selecting a risk cohort would arise in the case of reactive arthritis or undifferentiated arthritis. Overall, early detection of SpA remains a challenging problem.

In summary, the currently available data on metabolomics in spondyloarthritis (SpA) provide new options for assessing disease activity and potentially evaluating cardiovascular risk.

## Data Availability

The datasets used and/or analysed during the current study are available from the corresponding author on reasonable request.
